# Cardiac metastasis in endometrial carcinoma: A rare case report

**DOI:** 10.1097/MD.0000000000046353

**Published:** 2026-05-12

**Authors:** Liyuan Zhang, Zhilong Chen, Lu Wang, Yilin Guo, Hu Zhao

**Affiliations:** a Department of Gynecology, The Second Affiliated Hospital of Zhengzhou University, Zhengzhou, Henan Province, China.

**Keywords:** cardiac metastasis, comprehensive treatment, endometrial carcinoma, immunotherapy, multidisciplinary team consultation

## Abstract

**Rationale:**

Endometrial carcinoma (EC) is a frequently occurring cancer in the female reproductive system. Nevertheless, the occurrence of cardiac metastasis in cases of EC is uncommon and represents a significant risk to women’s health.

**Patient concerns:**

A 58-year-old female patient experienced abnormal vaginal bleeding for over 8 months and was eventually diagnosed with stage IVC (FIGO 2023) endometrial cancer characterized by mismatch repair deficiency that had spread to the heart.

**Diagnoses:**

Stage IVC (FIGO 2023) mismatch repair deficiency EC with cardiac metastasis.

**Interventions:**

After a thorough discussion with the multidisciplinary team, a detailed noninvasive treatment strategy was established: the initial phase involved 6 cycles of induction therapy using a combination of paclitaxel and carboplatin along with tislelizumab, resulting in a partial response. This was followed by 3 cycles of maintenance therapy with tislelizumab as a standalone treatment.

**Outcomes:**

Following the maintenance treatment, the cardiac metastasis was entirely eliminated. The patient experienced a progression-free survival duration of 9 months.

**Lessons:**

For patients with EC who have metastasized to rare sites such as the heart, comprehensive management guided by multidisciplinary team consultation is crucial, as it can significantly prolong survival.

## 1. Introduction

Endometrial carcinoma (EC) ranks among the most common cancers affecting women and has been experiencing an increase in both incidence and mortality rates worldwide,^[1]^ representing a serious risk to female health. Although advanced stages of EC often spread to organs such as the lungs, liver, bones, and brain,^[2]^ instances of cardiac involvement are extremely rare. Research covering the years from 1984 to 2019 indicated that cardiac metastasis occurs in 4.71% of all solid tumors, with those linked to EC being particularly infrequent.^[3]^ Importantly, the mismatch repair deficiency (MMRd) molecular subtype of EC, noted for its elevated tumor mutational burden, has emerged as a significant indicator of how well patients respond to immune checkpoint inhibitors (ICIs), thereby transforming treatment strategies for advanced cases.^[4]^ This report presents an unusually rare instance of stage IVC (FIGO 2023) high-grade MMRd EC with cardiac metastasis, where the patient experienced a positive treatment outcome through the collaboration of a multidisciplinary team (MDT) and thorough management of care.

## 2. Case presentation

**Figure 1.**
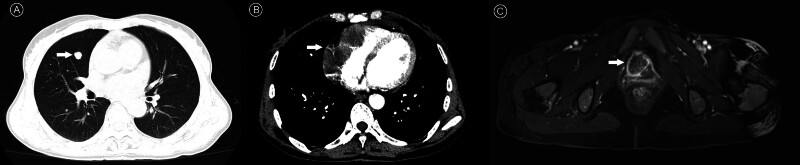
Baseline imaging evaluation of pulmonary, cardiac, and urethral involvement prior to treatment (May 22, 2024). (A) Chest CT shows a hyperdense pulmonary metastasis (arrow). (B) Cardiac CT reveals a hypodense cardiac metastasis (arrow). (C) Pelvic MRI (contrast-enhanced T1-weighted) demonstrates an enhancing urethral metastasis (arrow) with perilesional soft tissue enhancement. CT = computed tomography, MRI = magnetic resonance imaging.

On May 12, 2024, a 58-year-old woman arrived at a local hospital, reporting irregular vaginal bleeding for the past 8 months. A pelvic ultrasound identified a hypoechoic nodule measuring 25 mm × 33 mm located in the right anterior urethra. Following this, she underwent a hysteroscopy along with a needle biopsy of the urethral mass. The histopathological analysis post-surgery revealed: malignant growth within the uterine cavity; invasive carcinoma in the urethral mass. She was then referred to our facility for additional assessment and treatment. Her medical background was largely unremarkable, with no notable comorbid conditions. Both her menstrual and obstetric histories were normal. Family history included a father who passed away from hepatocellular carcinoma (exact age unknown) and a mother who died from pancreatic head carcinoma (exact age unknown). She has 3 older brothers, 1 younger brother, and a younger sister, all reported to be healthy. Upon admission, her physical examination indicated an Eastern Cooperative Oncology Group Performance Status of 1. The patient was alert and responsive but exhibited moderate cachexia. A gynecological examination revealed bloodstained vaginal discharge and a tender, poorly mobile mass (approximately 2 cm) in the anterior vaginal wall. The uterus was noted to be enlarged with mild tenderness, while the adnexal regions showed no significant issues. Cardiac assessment classified her as New York Heart Association class I. Pelvic ultrasound showed a heterogeneous echoic lesion (53 mm × 21 mm) in the uterine cavity with poorly defined myometrial borders, and an irregular hypoechoic nodule (30 mm × 22 mm) anterior to the vagina. Thoracoabdominal CT scans indicated multiple bilateral pulmonary solid nodules (suggestive of metastasis, clinical correlation recommended), pericardial effusion, and abnormal hyperdense areas in the right cardiac chambers (suspected neoplasm). Pelvic MRI revealed multiple uterine masses consistent with stage IV endometrial cancer, a nodular lesion anterior to the right urethra (likely metastasis), and enlarged lymph nodes in the pelvic and inguinal regions. An echocardiogram showed a hypoechoic mass (78 mm × 38 mm) attached to the walls of the right atrium and ventricle with mild protrusion into the cavity; tricuspid valve function was preserved (ejection fraction: 54%) (Fig. 1). The ECG showed no significant abnormalities. The pathological diagnosis indicated high-grade endometrioid carcinoma with focal clear cell differentiation (10%). Immunohistochemical results were as follows: CK(+), PAX-8 (partial+), ER (90%+), partial response (PR; 90%+), Ki-67 (70%+ hotspot), P53 (suspected mutant pattern), β-catenin (nuclear/cytoplasmic+). Mismatch repair status showed MLH1/MSH2(+) and MSH6/PMS2(−). Programmed death-ligand 1 GPS was 20, and HER-2 (Roche 4B5) was 1+. Molecular profiling (NGS) indicated an microsatellite instability-high (MSI-H)/MMRd subtype. Due to the costs associated with self-funded testing, the patient opted out of germline genetic testing. Tumor markers indicated an elevated CA125 level of 120 U/mL. Laboratory tests, including complete blood count, liver and kidney function, coagulation profile, thyroid function, and infectious disease screening, were all within normal ranges.

**Figure 2.**
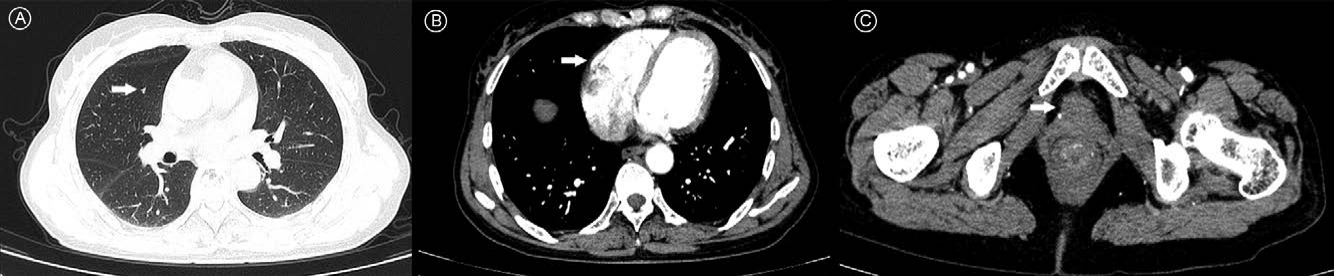
Posttreatment imaging evaluation of pulmonary, cardiac, and urethral involvement following 6 cycles of “paclitaxel + carboplatin + tislelizumab” therapy (November 12, 2024). (A) Chest CT demonstrates marked regression of the pulmonary metastasis (arrow). (B) Cardiac CT shows near-total resolution of the myocardial metastasis (arrow). (C) Pelvic CT shows resolution of the urethral metastatic focus (arrow) with residual periturethral stranding. CT = computed tomography.

**Figure 3.**
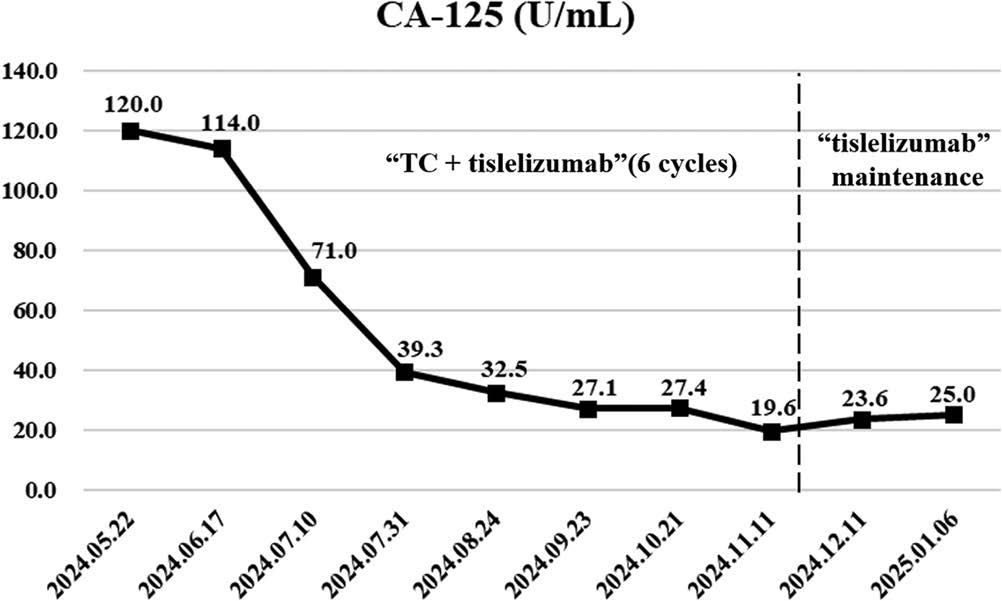
Serial CA-125 levels during the treatment course. Line graph demonstrates marked decline of serum CA-125 from 120.0 to 25.0 U/mL during treatment. Dashed vertical line indicates transition from 6 cycles of paclitaxel-carboplatin (TC) combined with tislelizumab to tislelizumab maintenance therapy. All values after August 2024 remain within normal limits (<35 U/mL). TC = paclitaxel-carboplatin.

After reviewing the patient’s medical background, imaging results, and pathological evidence, the diagnosis was confirmed as stage IVC MMRd EC with additional cancers affecting the heart, lungs, and vagina. Following a MDT discussion, the patient began a treatment plan that combined chemotherapy and immunotherapy. Between May and October 2024, the patient underwent 6 cycles of paclitaxel (175 mg/m²), carboplatin (AUC 5), and tislelizumab (200 mg every 3 weeks). During this period, the patient experienced grade 3 thrombocytopenia, which was addressed through the use of thrombopoietin receptor agonists and adjustments to the medication dosage. Imaging conducted in November 2024 showed that the heterogeneous echoes in the uterus had resolved, the urethral hypoechoic lesion had decreased to 13 mm × 7 mm, and the right cardiac mass had vanished. A thoracoabdominal CT scan indicated partial regression of metastases in the lungs, urethra, and left iliac vessels, categorized as a PR (Fig. 2). From November 2024 to January 2025, the patient received maintenance therapy with tislelizumab alone (200 mg every 3 weeks). A follow-up pelvic ultrasound in January 2025 confirmed the continued resolution of pelvic lesions, while echocardiography indicated stable thickening of the aortic valve without any functional issues. Throughout the treatment, the side effects related to chemotherapy and immunotherapy were carefully monitored, including regular checks of complete blood counts, tumor markers (with CA125 decreasing from 120 to 25 U/mL; Fig. 3), pelvic and cardiac ultrasounds, and thoracoabdominal CT scans. Management of hematologic toxicities and other side effects was prioritized to ensure a balance between treatment effectiveness and patient safety, with the goal of extending survival while maintaining quality of life. As of February 2025, the patient is in good overall health with stable disease management. The patient achieved a progression-free survival (PFS) of 9 months and reported no adverse events during the treatment (Table 1). This report illustrates the successful disease management attained during this initial treatment phase. This outcome highlights the significant effectiveness of the described chemoimmunotherapy approach.

**Table 1:** Timeline of clinical course.

Time	Stage	Key diagnostic findings/interventions
May 2024	Diagnosis	Stage IVC MMRd EC diagnosed with metastases to heart, lung, and urethra
May–October 2024	Induction	6 cycles of paclitaxel + carboplatin + tislelizumab
November 2024	Assessment	Imaging-confirmed PR
November 2024–January 2025	Maintenance	3 cycles of tislelizumab
February 2025	Outcome	Resolution of cardiac mass; PFS of 9 mo

EC = endometrial carcinoma, MMRd = mismatch repair-deficient, PFS = progression-free survival, PR = partial response.

## 3. Discussion

This case study focuses on a 58-year-old individual diagnosed with stage IVC MMRd EC, which was complicated by metastasis to the heart. Upon initial evaluation, the patient exhibited moderate cachexia, a sign indicative of systemic inflammation and a catabolic condition that correlates with reduced survival rates in cancer patients.^[5]^ In light of these significant risk factors, a thorough nonsurgical treatment strategy was devised after a MDT consultation. The patient underwent 6 cycles of induction therapy using a combination of paclitaxel and carboplatin along with tislelizumab, achieving PR. This was followed by 3 cycles of maintenance therapy with tislelizumab alone. Post-maintenance, the cardiac metastasis was completely resolved, and the patient achieved a PFS of 9 months.

Contemporary diagnostic methods combine molecular classification with conventional histopathology, dividing EC into 4 distinct subtypes: POLE-mutated, MMRd, no specific molecular profile, and p53-abnormal. This classification is crucial for understanding prognosis and treatment options.^[6]^ Molecular subtyping also informs adjuvant therapy decisions and highlights potential targets for precision medicine.^[7]^ Notably the identification of MMRd tumors as highly responsive to ICIs, which has reshaped the therapeutic paradigm for advanced disease.^[8]^ Cardiac metastases can be identified using imaging techniques like transthoracic echocardiography, contrast-enhanced CT, and cardiac MRI, which can show structural changes. Although pathological confirmation of the cardiac lesion was not obtained, which constitutes a limitation of this report, its concordant regression with confirmed extra-cardiac metastases following systemic therapy provides strong clinical evidence supporting the diagnosis of metastatic carcinoma. Nonetheless, potential differential diagnoses such as a cardiac thrombus or a benign cardiac mass cannot be entirely ruled out in the absence of a histological diagnosis. The MDT meeting was arranged for the patient, where the chief physician of cardiothoracic surgery observed imaging results indicating solid masses in the right atrium and ventricle. Echocardiography revealed a preserved left ventricular ejection fraction of 54% and no significant wall motion issues. The cardiac involvement represented extrinsic compression with patent outflow tracts and no severe arrhythmias. Given prohibitively high surgical risks, aggressive management of the primary malignancy with close cardiac surveillance was recommended. The pathologist emphasized molecular profiling identified MSI-H status in this patient. Immunohistochemistry for MMR proteins showed retained MLH1 and MSH2 expression, with a partial loss of MSH6 and a complete absence of PMS2, confirming the MMRd subtype. The PMS2 gene locus likely harbors a pathogenic mutation. Although the family history included paternal hepatocellular carcinoma (non-Lynch-associated) and maternal pancreatic head carcinoma (secondary Lynch-associated), germline verification was deferred due to undocumented age of onset and patient refusal of self-funded genetic testing – a decision not precluding immunotherapy selection.

The use of paclitaxel in conjunction with carboplatin serves as the primary treatment for advanced or recurrent EC.^[9]^ While carboplatin exhibits minimal cardiotoxicity, prolonged use of this regimen may induce myocardial dysfunction, necessitating cardiovascular risk monitoring during and posttreatment. Studies demonstrate that MMRd ECs frequently correlate with adverse prognostic factors, including high histological grade and lymphovascular space invasion.^[10]^ Recent phase III trials have confirmed the effectiveness of adding immunotherapy to the paclitaxel and carboplatin regimen, with a meta-analysis showing significant enhancements in PFS for patients with MMRd tumors.^[11]^ The medical oncologist emphasized that Chinese guidelines for EC recommend ICIs for MMRd subtypes. Although pembrolizumab is a guideline-preferred ICI, its substantial cost often limits accessibility for patients requiring self-paid medication. The decision to use tislelizumab was based on a compelling rationale integrating mechanistic superiority, robust clinical data, and regional healthcare economics. Mechanistically, tislelizumab is differentiated by its engineered Fc domain, which is designed to minimize binding to FcγR on macrophages. This is in direct contrast to pembrolizumab, which possesses a conventional IgG4 Fc region with intact FcγR binding capacity. Preclinical studies have demonstrated that FcγR binding by anti-programmed cell death protein 1 antibodies can induce antibody–dependent phagocytosis of T-cells by tumor-associated macrophages, thereby potentially counteracting the intended T-cell-mediated antitumor immunity. By mitigating this negative regulatory pathway, tislelizumab’s Fc-engineered design aims to preserve effector T-cells in the tumor microenvironment.^[12]^ Clinically, trials registered on ClinicalTrials.gov (NCT-registered) confirm its efficacy in Chinese MSI-H/MMRd solid tumors.^[13]^ Critically, from a regional healthcare context, tislelizumab is included in the Chinese National Reimbursement Drug List for relevant indications, whereas pembrolizumab for this specific context may not be. This provided a decisive advantage in ensuring long-term treatment affordability and accessibility for our patient, making a sustained and effective therapy feasible. For this patient, a comprehensive nonsurgical treatment plan was implemented following multidisciplinary consultation: induction therapy with paclitaxel/carboplatin combined with tislelizumab for 6 cycles, achieving PR. Subsequently, maintenance therapy with single-agent tislelizumab was administered for 3 cycles. After the maintenance therapy, the cardiac metastasis completely resolved. The patient achieved a PFS of 9 months. This integrated strategy effectively controlled tumor progression, extended survival, and preserved quality of life.

Cardiac metastases from EC carry a grave prognosis, with a median survival often not exceeding 6 months.^[14,15]^ Traditional management has relied on high-risk local interventions. For instance, long-term survival was achieved by Bigsby et al through debulking surgery combined with cardiac radiation and liposomal doxorubicin, though the patient ultimately succumbed to radiation-induced constrictive pericarditis.^[16]^ Similarly, Cipullo et al and Erturk et al employed sternotomy-based resection, with the latter incorporating pembrolizumab.^[17,18]^ To our knowledge, this is the first documented case of complete radiographic resolution of a cardiac metastasis from EC using a purely systemic, nonsurgical approach. This was achieved with a chemoimmunotherapy regimen of paclitaxel, carboplatin, and tislelizumab, offering a novel and effective therapeutic avenue for patients who are not candidates for or wish to avoid high-risk invasive procedures. A direct comparison with key reported cases underscores this distinction (Table 2).

**Table 2:** Comparison of therapeutic strategies and outcomes in reported cases of cardiac metastasis from endometrial carcinoma.

Case	Primary treatment	Systemic agent(s)	Key outcome
Bigsby et al^[16]^	Surgery + radiotherapy + chemotherapy	Liposomal doxorubicin, cisplatin	6-yr survival; death from radiation toxicity
Cipullo et al^[17]^	Surgery + radiotherapy	Not specified (adjuvant)	Disease control via invasive local therapy
Erturk et al^[18]^	Surgery + chemoimmunotherapy	Paclitaxel, carboplatin, pembrolizumab	Sustained control; combined surgical and systemic approach
Present case	Exclusively systemic nonsurgical	Paclitaxel, carboplatin, tislelizumab	Complete radiographic resolution without surgery or radiation; PFS of 9 mo

PFS = progression-free survival.

The remarkable response achieved in our case, including the complete resolution of a cardiac metastasis, stands in stark contrast to the therapeutic challenges posed by other uterine malignancies, such as uterine leiomyosarcoma (uLMS). Unlike MMRd ECs, uLMS is characterized by a low tumor mutational burden and a genomic landscape dominated by the inactivation of tumor suppressor genes such as TP53 and RB1, which may underlie its reported resistance to immunotherapy.^[19]^ Moreover, the clinical benefit of systemic therapy in uLMS remains limited. Recent meta-analyses have concluded that adjuvant chemotherapy did not significantly reduce recurrence rates in early-stage disease.^[20,21]^ This dichotomy highlights the critical importance of molecular subtyping in guiding treatment selection for uterine cancers. It underscores that for specific subsets, such as MMRd EC, immunotherapy can achieve unprecedented efficacy, whereas for others like uLMS, the development of effective novel therapies remains a pressing, unmet need.

Despite rapid clearance of the cardiac metastasis achieved in this case, advanced MMRd EC carries an inherent high-risk of recurrence. Future investigations should prioritize optimization of immunotherapy maintenance strategies, such as combination with antiangiogenic agents. For progressive MMRd endometrial cancer beyond immunotherapy, emerging antibody-drug conjugates targeting novel antigens including TROP-2, HER2, and FRα are under active clinical development. These approaches represent promising alternatives for future therapeutic selection.

The patient’s personal perspective or subjective experiences were not documented, as the clinical focus centered on the cardiac metastasis in MMRd EC. All management decisions were made with informed consent.

## 4. Conclusion

In managing EC with cardiac metastasis, clinicians should initiate a MDT consultation to holistically evaluate systemic performance status, assess cardiac functional compromise from metastatic burden, and delineate disease extent. Risk-benefit stratification integrating comprehensive genomic profiling (such as MSI/MMR status and pan-cancer targets) is essential for formulating individualized therapeutic regimens. Implementation of longitudinal surveillance protocols with dynamic response assessment enables timely intervention adjustment, ultimately optimizing both quality of life and survival outcomes.

## Author contributions

**Conceptualization**: Liyuan Zhang, Zhilong Chen.

**Data curation**: Liyuan Zhang, Lu Wang.

**Formal analysis**: Lu Wang, Yilin Guo.

**Funding acquisition**: Zhilong Chen.

**Methodology**: Liyuan Zhang, Zhilong Chen, Hu Zhao.

**Project administration**: Zhilong Chen, Hu Zhao.

**Writing – original draft**: Liyuan Zhang.

**Writing – review & editing**: Zhilong Chen.
